# Comparative Metabolome and Transcriptome Analyses Reveal Differential Enrichment of Metabolites with Age in *Panax notoginseng* Roots

**DOI:** 10.3390/plants13111441

**Published:** 2024-05-23

**Authors:** Xinru Yan, Ao Zhang, Yiming Guan, Jinlong Jiao, Murad Ghanim, Yayu Zhang, Xiahong He, Rui Shi

**Affiliations:** 1 Yunnan Provincial Key Laboratory for Conservation and Utilization of In-Forest Resource, International Ecological Forestry Research Center of Kunming, Southwest Forestry University, Kunming 650224, China; yan874711549@swfu.edu.cn (X.Y.); ao2040749351@swfu.edu.cn (A.Z.); jinlong@swfu.edu.cn (J.J.); 2 Institute of Special Wild Economic Animal and Plant Science, Chinese Academy of Agricultural Sciences, Changchun 130112, China; gymcau@126.com; 3 Department of Entomology, Institute of Plant Protection, 68 Hamaccabim Road, Rishon LeZion 7505101, Israel; ghanim@agri.gov.il

**Keywords:** *Panax notoginseng*, gene expression, differential metabolites, therapeutic use

## Abstract

*Panax notoginseng* is a perennial plant well known for its versatile medicinal properties, including hepatoprotective, antioxidant, anti-inflammatory, anti-tumor, estrogen-like, and antidepressant characteristics. It has been reported that plant age affects the quality of *P. notoginseng*. This study aimed to explore the differential metabolome and transcriptome of 2-year (PN2) and 3-year-old (PN3) *P. notoginseng* plant root samples. Principal component analysis of metabolome and transcriptome data revealed major differences between the two groups (PN2 vs. PN3). A total of 1813 metabolites and 28,587 genes were detected in this study, of which 255 metabolites and 3141 genes were found to be differential (*p* < 0.05) between PN2 vs. PN3, respectively. Among differential metabolites and genes, 155 metabolites and 1217 genes were up-regulated, while 100 metabolites and 1924 genes were down-regulated. The KEGG pathway analysis revealed differentially enriched metabolites belonging to class lipids (“13S-hydroperoxy-9Z, 11E-octadecadionic acid”, “9S-hydroxy-10E, 12Z-octadecadionic acid”, “9S-oxo-10E, 12Z-octadecadionic acid”, and “9,10,13-trihydroxy-11-octadecadionic acid”), nucleotides and derivatives (guanine and cytidine), and phenolic acids (chlorogenic acid) were found to be enriched (*p* < 0.05) in PN3 compared to PN2. Further, these differentially enriched metabolites were found to be significantly (*p* < 0.05) regulated via linoleic acid metabolism, nucleotide metabolism, plant hormone signal transduction, and arachidonic acid metabolism pathways. Furthermore, the transcriptome analysis showed the up-regulation of key genes *MAT*, *DMAS*, *SDH*, gallate 1-beta-glucosyltransferase, and beta-D-glucosidase in various plants’ secondary metabolic pathways and *SAUR*, *GID1*, *PP2C*, *ETR*, *CTR1*, *EBF1/2*, and *ERF1/2* genes observed in phytohormone signal transduction pathway that is involved in plant growth and development, and protection against the various stressors. This study concluded that the roots of a 3-year-old *P. notoginseng* plant have better metabolome and transcriptome profiles compared to a 2-year-old plant with importantly enriched metabolites and genes in pathways related to metabolism, plant hormone signal transduction, and various biological processes. These findings provide insights into the plant’s dynamic biochemical and molecular changes during its growth that have several implications regarding its therapeutic use.

## 1. Introduction

*Panax notoginseng* is a perennial plant well known for its versatile medicinal properties, including hepatoprotective, antioxidant, anti-inflammatory, anti-tumor, estrogen-like, and antidepressant characteristics [1,2]. *Panax notoginseng* is a traditional Chinese herbal medicine primarily grown in the Yunnan Province of Southwest China [3,4]. It is an increasingly popular choice in the health industry because it is commonly used as a primary constituent in capsules, tablets, pills, and medicinal liquors [5]. It contains a diverse array of healthy nutritional components such as saponins, flavonoids, amino acids, volatile oils, plant-based alcohols, inorganic salts, inorganic ions, and many other active ingredients that exhibit extensive clinical applications and pharmacological effects [6]. Currently, *P. notoginseng* roots are used in medical practices to treat atherosclerosis, cardiac angina, apoplexy, and coronary heart disease [7]. For a very long time, *P. notoginseng*’s root has been essential to Chinese medicine. It is the primary component of Yun Nan Bai Yao, a well-known herbal hemostatic treatment that reduces inflammation, stops bleeding, and relieves pain [5].

Studies revealed that mature *P. notoginseng* plants are comparatively more expensive than younger plants because of their higher quality [5]. Further, three-year-old plants are primarily used as medicinal resources, while two-year-old plants are also sold in the pharmaceutical industry. *P. notoginseng* plant parts and developmental stages can affect the safe application of medicinal products [1]. So, it is essential to comprehend the age-related variations in *P. notoginseng* metabolites to ensure the safe use of pharmaceutical product ingredients.

The metabolomics technique offers a thorough analysis of plant metabolites, revealing comprehensive insights into the distinct metabolomic profile exhibited by plants. This methodology expedites the process of pinpointing putative indicators for the analysis of metabolomic variations in herbal medicines derived from various congeneric species [8], plant parts [9], collection regions [10], processing techniques [11], or varying growing ages [12]. High-throughput analytical methods, including DNA sequencing [13], gas chromatography–mass spectrometry (GC-MS) [14], and high-performance liquid chromatography–mass spectrometry (HPLC-MS) [1] have been applied to Chinese herbal medicine for their variety classification and origin identification. Compared to other tissues, ginseng roots, which are the primary medicinal component, have drawn a lot of interest in terms of different genes and pathways involved in the formation of various metabolites. When comparing the content of ginsenoside from one to thirteen years of produced ginseng, it was found that the amount of ginsenoside increased with the number of culture years [15].

Using HPLC-MS to measure the ginsenoside content of farmed ginseng from 1 to 6 years old, it was found that the cultivated ginseng at 5 and 6 years old had a high Ro/Re ratio, whereas the cultivated ginseng at 2 and 3 years old had a lower ratio [16]. Transcriptome sequencing has been utilized to pinpoint functional genes associated with the production of plant-active ingredients [17]. The molecular basis of the synthetic regulatory networks of the corresponding secondary metabolites is obtained by investigating synthesis pathways and examining the control of gene expression [18]. In a previous study, transcriptome sequencing on the roots, stems, and leaves of cultivated ginseng and wild ginseng with different growth years was performed that revealed growth years had significant effects on the genes related to ginsenoside synthesis in cultivated ginseng, and the effects were different in the roots, stems and leaves [19]. Another study revealed that the expression of genes involved in the triterpene skeleton and the downstream ginsenoside synthesis pathway in 6-year-old ginseng was higher than in 1-year-old ginseng, and genes in 1-year-old ginseng were more highly expressed in the mevalonic acid (MVA) and 2-C-methyl-D-erythritol-4-phosphate (MEP) synthesis pathways [20]. All these studies indicate that different growth years have different metabolome and transcriptome expression profiles.

On account of previous studies, we hypothesized that the 3-year-old *P. notoginseng* plant might have better metabolomic and transcriptome expression profiles compared to the 2-year-old plant, which could have therapeutic importance. For this, we used non-targeted HPLC and RNA sequencing techniques, respectively, to give insight into the differentially enriched metabolites, genes, and their pathways. 

## 2. Materials and Methods

### 2.1. Plant Material and Tissue Collection

For the current study, root tissue samples of 2- and 3-year-old *P. notoginseng* (Burgill) plants were collected at Linxia organic *P. notoginseng* planting base, Zhutang Township, Lancang Lahu Autonomous County, Pu’er City, Yunnan Province (22.42° N, 99.50° E, 1510 m). A total of six samples were collected, with each treatment having three replicates: a 2-year-old *P. notoginseng* plant (PN2): PN2-1, PN2-2, and PN2-3; and 3-years-old *P. notoginseng* plants (PN3): PN3-1, PN3-2, and PN3-3. The samples were collected from the healthy plants’ root tissues and were carried out to the lab into frozen liquid nitrogen and then stored at −80 °C before use.

### 2.2. Metabolome Analysis

To extract metabolites for the metabolomic study, 100 mg of root tissue was crushed separately with liquid nitrogen. After that, the homogenate was once again vortexed in a solution containing 0.1% formic acid and 80% chilled methanol. The samples were centrifuged for five minutes at 15,000 rpm and 4 °C after being incubated on ice for five minutes. The supernatant was then treated with UHPLC-MS/MS grade water to 53% methanol concentration. After that, the samples were transferred to a fresh Eppendorf tube and centrifuged for 10 min at 4 °C and 15,000× *g*. A Vanquish UHPLC system (Thermo Fisher, Karlsruhe, Germany) was used in conjunction with an Orbitrap Q ExactiveTM HF-X mass spectrometer (Thermo Fisher, Germany). The samples were injected onto a Hypesil Gold column (100 mm × 2.1 mm, 1.9 µm) over a 17 min linear gradient at a flow rate of 0.2 mL/min. Eluent A (0.1% formic acid in water) and eluent B (methanol) were used in the positive polarity mode, whereas eluent A (5 mM ammonium acetate, pH 9.0) and eluent B (methanol) were used in the negative polarity mode. The solvent gradient was as follows: 2% B, 1.5 min; 2–100% B, 12.0 min; 100% B, 14.0 min; 100–2% B, 14.1 min; 2% B, 17 min. A positive/negative polarity Q ExactiveTM HF-X mass spectrometer with a spray voltage of 3.2 kV, capillary temperature of 320 °C, sheath gas flow rate of 40 arb, and aux gas flow rate of 10 arb was used.

The raw data files obtained by the UHPLC-MS/MS were processed using Compound Discoverer 3.1 (CD3.1, Thermo Fisher) to perform peak alignment, peak selection, and quantification for each metabolite. The following key parameters were set: a retention time tolerance of 0.2 min, an actual mass tolerance of 5 ppm, a signal intensity tolerance of 30%, a signal/noise ratio of 3, and a minimum intensity threshold of 100,000. The peak intensities were then normalized in relation to the overall spectral intensity. After normalizing the data, it was used to forecast molecular formulae based on additive ions, molecular ion peaks, and fragment ions. Peaks were then cross-referenced against the mzCloud, mzVault, and MassList databases to obtain exact qualitative and relative quantitative findings.

### 2.3. Transcriptome Analysis

The RNeasy Plant Mini Kit (Qiagen, Seoul, Republic of Korea) was used to extract total RNA from 50 mg of each root sample in accordance with the manufacturer’s instructions. Utilizing 1% agarose gels and a Nano Photometer spectrophotometer (IMPLEN, Los Angeles, CA, USA), the purity of the isolated RNAs was assessed. Quantification of RNA was carried out using a Qubit RNA Assay Kit in a Qubit 2.0 Fluorometer (Life Technologies, Carlsbad, CA, USA). Additionally, RNA integrity was tested using the Agilent Bioanalyzer 2100 system’s RNA Nano 6000 Assay Kit (Agilent Technologies, Santa Clara, CA, USA). Following the manufacturer’s instructions [21], sequencing libraries were made using the NEB Next Ultra RNA Library Prep Kit, and the libraries were sequenced on an Illumina HiSeq 2000 platform.

Custom Perl scripts were first utilized to preprocess raw data in fastq format in order to prepare it for the sequenced data analyses [22]. A specific length range was then selected from the clean reads to be used in all downstream analysis. The genome website was immediately accessed to retrieve the gene model annotation files and reference genome (http://www.herbal-genome.cn, accessed on 22 August 2023) [23]. STAR (v.2.5.1b) was used to create a reference genome index and to align paired-end clean reads to the reference genome. For junction readings, STAR used the maximum mappable prefix (MMP) technique to ensure precise mapping output. HTSeq (v.0.6.0) was used to count the number of reads mapped to each gene. Then, using the length of the gene and the number of mapped reads, the estimated number of fragments per kilobase of transcripts per million mapped reads (FPKM) for each gene was calculated.

### 2.4. Statistical Analysis

Principal component analysis (PCA) was carried out using SIMCA-P 14.1 (Umetrics, Umea, Sweden). Pearson correlation coefficients (PCC) among samples were computed using the “cor” function in R, and the results were visualized as heatmaps. Differential metabolites were defined as those with a VIP > 1, a *p*-value < 0.05, and a fold change (FC) >2. Following that, the KOBAS 2.0 program was used to perform KEGG enrichment analysis on the differentially accumulated metabolites [24]. For the purpose of performing differential expression analysis between the two groups, the DESeq2 R package (v.1.10.1). Benjamini and Hochberg’s approach was applied to the resultant *p* values in order to regulate the false discovery rate. Gene Ontology (GO) enrichment analysis was used to analyze Differentially Expressed Genes (DEGs) by adjusting for gene length bias using the Wallenius noncentral hypergeometric distribution inside GOseq [25].

## 3. Results

### 3.1. Metabolome of P. notoginseng (PN2 vs. PN3)

#### 3.1.1. Differential Metabolites

**Figure 1:**
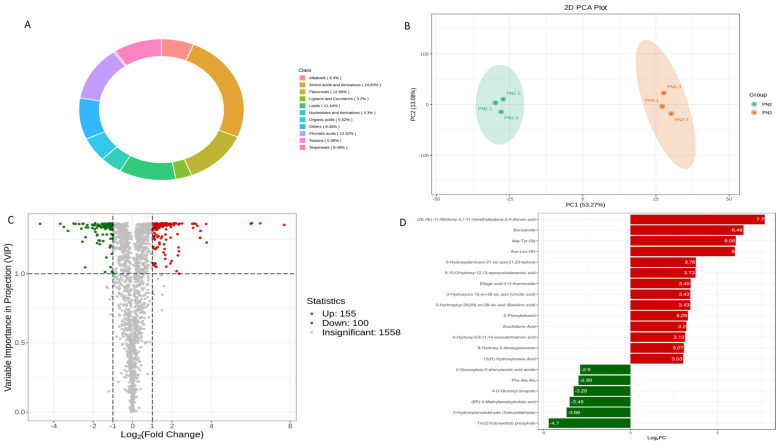
Metabolome analysis of *P. notoginseng* (**A**) Metabolite categories make up a donut chart. Each color represents a metabolite class, and the patch area represents the proportion of that category (**B**) PCA plot, each point in the graph represents a sample, samples in the same group are represented by the same color (**C**) Volcano plot, each dot in the volcano diagram represents a metabolite, where the green dot represents the down-regulated differential metabolite, the red dot represents the up-regulated differential metabolite, and the gray dot represents the detected but not significantly differentiated metabolite and (**D**) FC_Bar_Graph, red represents an upward adjustment of metabolite content, and green represents a downward adjustment of metabolite content.

**Figure 2:**
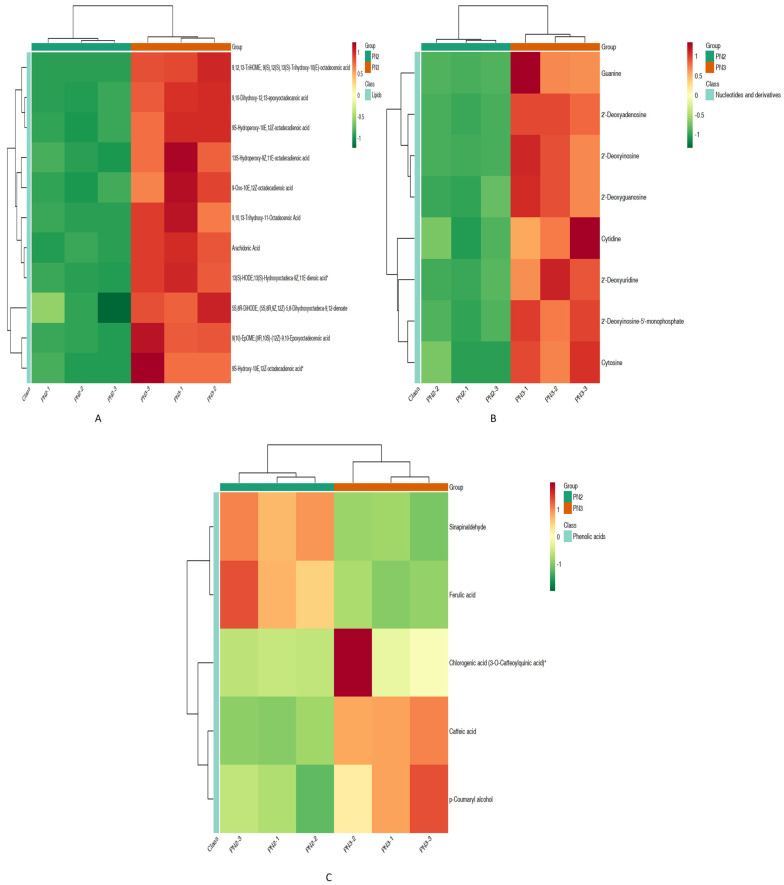
KEGG database differential metabolites enrichment heatmap of different classes: (**A**) lipids, (**B**) nucleotide and derivatives, and (**C**) phenolic acids.

A total of 1813 metabolites were detected in this study. The donut chart showed these metabolites belonged to 11 major groups of metabolites (Figure 1A). The highest number of detected metabolites were related to class amino acids and derivatives (24.93%), followed by mainly flavonoids (12.69%), phenolic acid (12.52%), lipids (11.14%) and terpenoids (9.49%). Further, the correlation observed between replicates was ≥0.92 (Supplementary Figure S1). Principal component analysis (PCA) of *P. notoginseng* revealed significant differences between PN2 vs. PN3 metabolic profiles, with PC1 showing a 53.54% variation, whereas PC2 showed a 13% variation (Figure 2A). Further, the grouping was also confirmed via clustering analysis (Supplementary Figure S2).

Of the total 1813 detected metabolites, 255 were significantly (*p* < 0.05) different between PN2 vs. PN3, as shown in the Volcano plot, whereas 1558 were non-significant (*p* > 0.05) (Figure 1C). Of the total significant metabolites, 155 were up-regulated, while 100 were down-regulated. Further, based on the quantitative information of each metabolite, the top 20 differential metabolites were arranged and are shown in the form of FC_Bar_Graph (Figure 1D). In top 20, the up-regulated differential metabolites detected were “(2E, 4E)-11-methoxy-3,7,11-trimethyldodeca-2,4-dienoic acid”, “Benzamide”, “Asp-Tyr-Gly”, “Asn-Leu-OH”, “3-Hydroxydammara-21-oic acid 21,23-lactone”, “9,10-Dihydroxy-12,13-epoxyoctadecanoic acid”, “Ellagic acid-4-O-rhamnoside”, “3-Hydroxyurs-12-en-28-oic acid (Ursolic acid)”, “3-Hydroxylup-20(29)-en-28-oic acid (Betulinic acid)”, “2-Phenylethanol”, “Arachidonic acid”, “5-Hydroxy 6,8,11,14-eicosatetraenoic acid”, “8-Hydroxy-2-deoxyguanosine”, and “15(R)-Hydroxylinoleic acid”, whereas, “2-Glucosyloxy-2-phenylacetic acid amide”, “Phe-Ala-Ala”, “4-O-glucosyl-sinapate”, “(6R)-5-Methyltetrahydrofolic acid”, “2-Hydroxybenzaldehyde (Salicylaldehyde)”, and “Tris(2-butoxyethyl) phosphate” were down-regulated.

#### 3.1.2. KEGG Differential Metabolites Enrichment Analysis (PN2 vs. PN3)

The KEGG heatmaps of differentially enriched metabolites from different classes were plotted (Figure 2A–C). The metabolites belonging to lipids class “13S-hydroperoxy-9Z, 11E-octadecadionic acid”, “9S-hydroxy-10E, 12Z-octadecadionic acid”, “9S-oxo-10E, 12Z-octadecadionic acid”, and “9,10,13-trihydroxy-11-octadecadionic acid” were found to be highly enriched in PN3 compared to PN2 (Figure 2A). Similarly, guanine and cytidine belonging to class nucleotides and derivatives and chlorogenic acid belonging to class phenolic acids were also found to be highly enriched in PN3 compared to PN2 (Figure 2B,C).

#### 3.1.3. KEGG Pathways Enrichment Analysis

**Figure 3:**
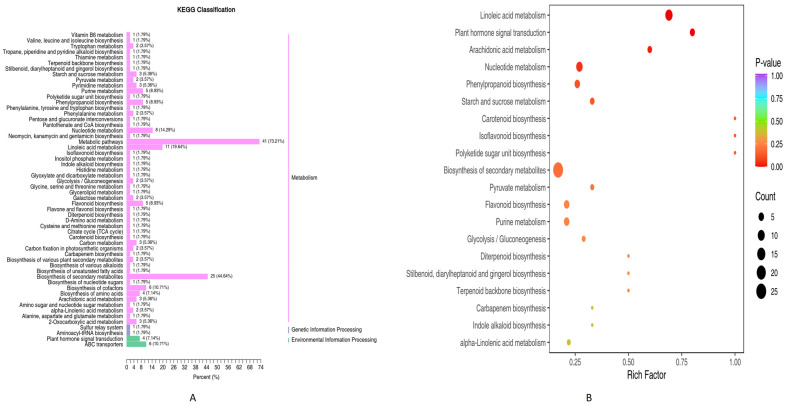
KEGG pathways analysis (**A**) Bar graph showing the KEGG database enriched pathways and classes and (**B**) Bubble diagram showing the significantly enriched pathways (*p* < 0.05) of metabolites.

The significantly differential metabolites (PN2 vs. PN3) detected above were classified according to the pathway categories in the KEGG database (Figure 3A). The metabolism category contained the highest number of enriched pathways including metabolic pathways 41 (73.21%), biosynthesis of secondary metabolites 25 (44.64%), Linoleic acid metabolism 11 (19.64%), nucleotide metabolism 8 (14.29%), and biosynthesis of cofactors 6 (10.71%) majorly, following the environmental processing category that included ABC transporters 6 (10.71%) and plant hormone signal transduction 4 (7.14%), and the genetic information processing category that included aminoacyl-tRNA biosynthesis 1 (1.79%) and sulfur relay system 1 (1.79%). Further, the KEGG pathways enrichment analysis was performed, and results are shown in the form of a Rich factor bubble plot (Figure 3B). Linoleic acid metabolism, nucleotide metabolism, plant hormone signal transduction, and Arachidonic acid metabolism pathways were found to be significantly enriched (*p* < 0.05) between both groups (PN2 vs. PN3).

### 3.2. Transcriptome of Panax notoginseng (PN2 vs. PN3)

#### Differentially Expressed Gene Analysis

**Figure 4:**
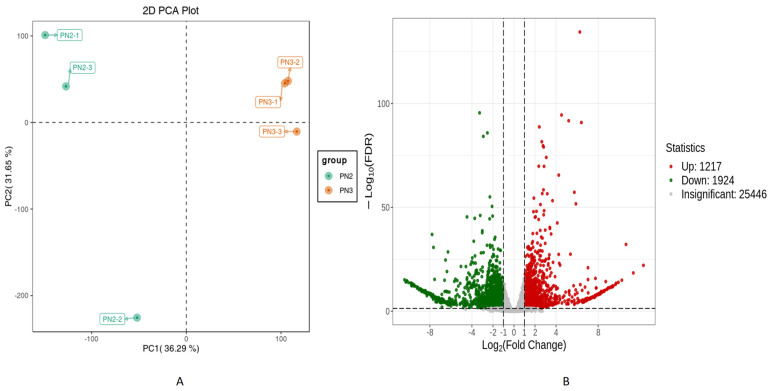
Transcriptome analysis of *P. notoginseng* (**A**) PCA plot, each point in the graph represents a sample, samples in the same group are represented by the same color (**B**) Volcano plot, each dot in the volcano diagram represents a gene, where the green dot represents the down-regulated differential genes, the red dot represents the up-regulated differential genes, and the gray dot represents the detected but not significantly differentiated genes.

The sequencing analysis of six libraries produced a total of 38.13 Gb clean data with an average of 6 Gb per sample. The Q30 base percentage, average sequencing error rate, and GC content were ≥94.08%, 0.03%, and 43.48%, respectively (Supplementary Table S1). The overall gene expression of the six libraries is presented in Supplementary Figure S3. The Pearson’s Correlation between the replicates was very high (0.92–1) (Supplementary Figure S4). The PCA plot of transcriptome sequencing data of *P. notoginseng* groups (PN2 vs. PN3) was drawn to similarities and differences between them (Figure 4A). PC1 revealed a 36.29% variance, whereas PC2 revealed 31.65% variance. PN2 vs. PN3 samples were grouped into their respective treatment, revealing differences in the transcriptome profiles. These results indicated the quality of data was good for further analysis. A total of 28,587 genes were found, of which 3141 were found to be significant (*p* < 0.05) between PN2 vs. PN3 (Figure 4B). Of the total 3141 significant differential genes, 1217 genes were up-regulated, while 1924 genes were down-regulated.

### 3.3. Gene Ontology Pathways Enrichment (GO) Analysis

**Figure 5:**
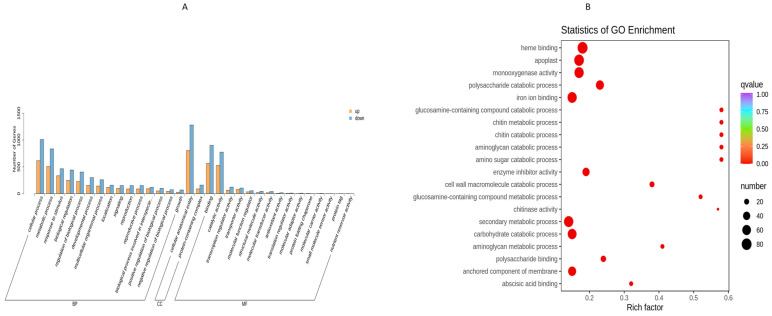
GO-enriched pathways analysis (**A**) Bar graph showing the up-regulated and down-regulated differentially enriched genes in GO terms and (**B**) Bubble diagram showing the GO-enriched pathways of differentially enriched genes.

The significantly differed up-regulated and down-regulated differential genes were classified using the gene ontology pathways database (Figure 5A). The biological processes category included the cellular processes, metabolic processes, response to stimulus, and biological regulation majorly; the category cellular component included cellular, anatomical entity, and protein-containing complex; and the category molecular functions included the binding and catalytic activity majorly. Further, the GO enrichment analysis was performed, and results are shown in the form of a Rich factor bubble plot (Figure 5B). Heme binding, apoplast, monooxygenase activity, polysaccharide catabolic process, iron binding, enzyme inhibitor activity, secondary metabolic process, carbohydrate catabolic process, and an anchored component of the membrane were found to be highly enriched (*p* < 0.05) pathways.

### 3.4. Combined Metabolome and Transcriptome KEGG Enrichment Analysis

**Figure 6:**
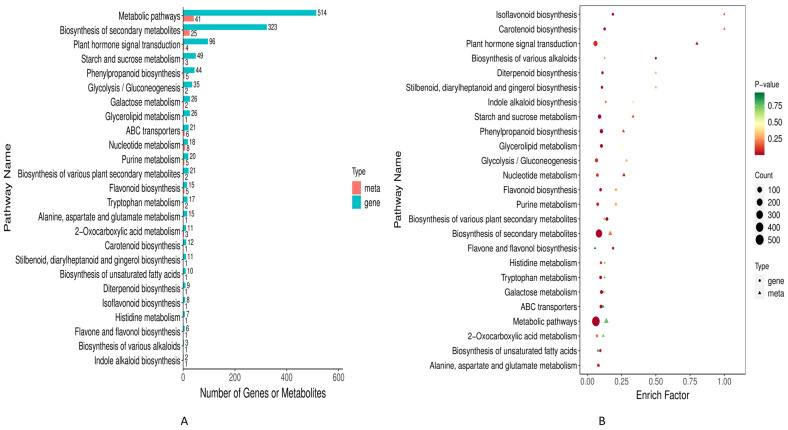
Combined KEGG pathways analysis of metabolome and transcriptome (**A**) Bar graph showing the KEGG database enriched pathways and (**B**) Bubble diagram showing the enriched pathways of metabolites.

The combined metabolome and transcriptome sequence data were analyzed in the KEGG database. A bar chart was drawn using the KEGG pathway co-enriched by the two omics, and the bar chart showed the number of differential metabolites and differential genes enriched into a pathway (Figure 6A). For both omics the highest number of genes and metabolites were found to be enriched in metabolic pathways (541 and 41), biosynthesis of secondary metabolites (323 and 25), and plant hormone signal transduction, respectively. Further, bubble rich factor diagram revealed isoflavonoid biosynthesis, carotenoid biosynthesis, plant hormone signal transduction, starch sucrose metabolism, phenylpropanoid biosynthesis, and nucleotide metabolism were found to be significantly (*p* > 0.05) co-enriched for both omics (Figure 6B).

### 3.5. Expression Changes in Various Plants Secondary Metabolic Pathways

**Figure 7:**
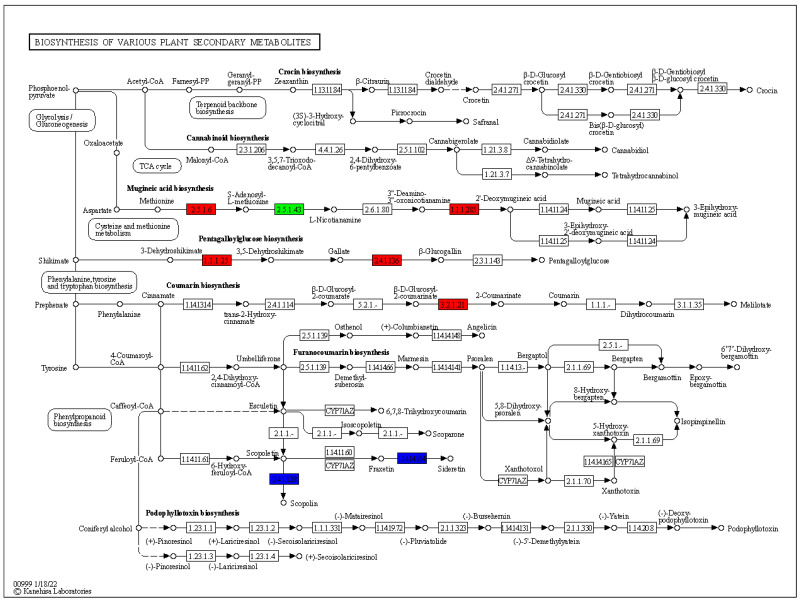
Pathway representation of various plants’ secondary metabolites and genes. The red color represents the up-regulation of genes, green represents the down-regulation of genes, while blue color represents no significant change in expression. Names in boxes represent genes.

The differential genes and differential metabolites of PN2 vs. PN3 were mapped to the KEGG pathway diagram at the same time so as to better understand the relationship between genes and metabolites. Notably, we observe significant changes in the expression of genes associated with various plants’ secondary metabolites (Figure 7). In the mugineinc acid biosynthesis pathway two genes S-Adenosylmethionine synthetase (EC 2.5.1.6), also known as methionine adenosyltransferase (*MAT*) and 3″-deamino-3″-oxonicotianamine reductase (*DMAS*) catalyzes were found to be up-regulated, whereas homospermidine synthase (spermidine-specific) (EC 2.5.1.45) was found down-regulated. For the biosynthesis of pentagalloylglucose, shikimate dehydrogenase (*SDH*) (EC 1.1.1.25) and gallate 1-beta-glucosyltransferase (EC 2.4.1.136) genes were found to be up-regulated in the shikimate pathway. Similarly, beta-D-glucosidase (EC 3.2.1.21) was found to be up-regulated in the coumarin biosynthesis pathway.

### 3.6. Expression Changes in Phytohormone Signaling Pathways

**Figure 8:**
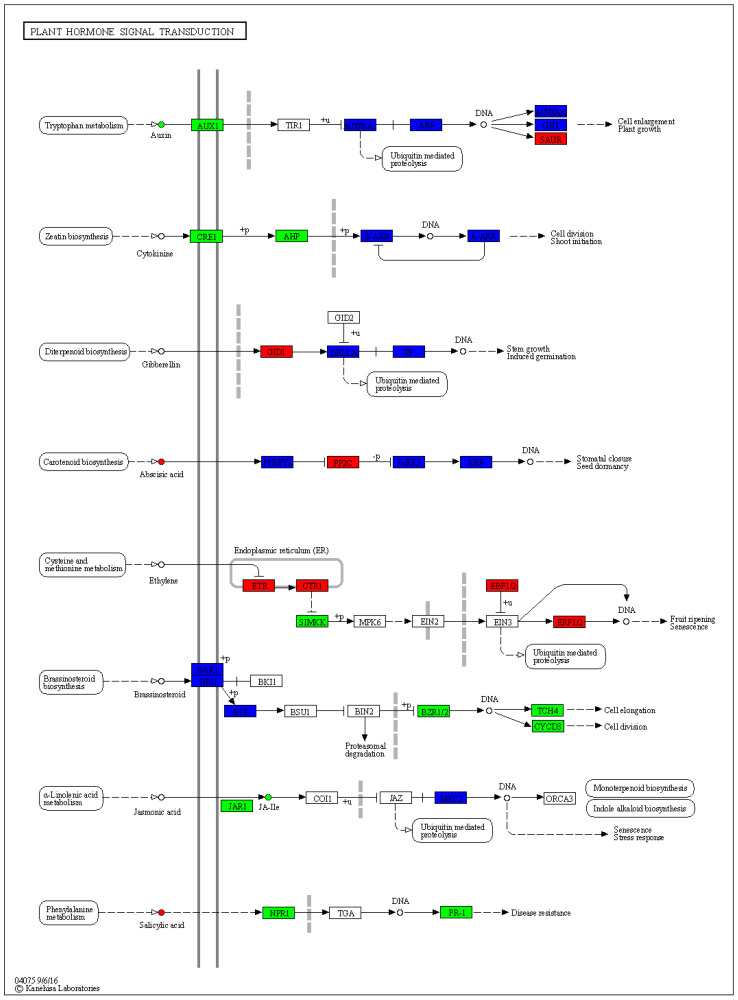
Pathway representation of plant hormone signal transduction pathway genes and metabolites. The red color represents the up-regulation of genes, green represents the down-regulation of genes, while blue color represents no significant change in expression. Names in boxes represent genes.

In phytohormone signaling pathways, we observe significant changes in the expression of genes associated with various phytohormones (Figure 8). In the auxin signaling pathway, which is involved in cell enlargement and plant growth, the *AUX1* was down-regulated, whereas *SAUR* was up-regulated. The *CRE1* and *AHP* were down-regulated in the cytokine signaling pathways that are involved in cell division and shoot initiation. In the gibberellin signaling pathway, *GID1* was up-regulated. Similarly, *PP2C* was up-regulated in the abscisic acid signaling pathway, and *ETR*, *CTR1*, *EBF1/2*, and *ERF1/2* were up-regulated, and *SIMKK* was down-regulated in the ethylene signaling pathway. In the brassinosteroid signaling pathway, *BZR1/2*, *TCH4*, and *CYCD3* were down-regulated. Further, JAR1 was down-regulated in the jasmonic acid signaling pathway, and *NPR1* and *PR-1* were down-regulated in salicylic acid signaling pathways.

## 4. Discussion

The current study was performed to reveal the differences in metabolome and transcriptome profiles of 2-year and 3-year-old *P. notoginseng* plants using its root tissue samples. Previous studies have explored the 2- and 3-year-old *P. notoginseng* plant metabolome and transcriptome especially targeting the saponins and found the 3-year-old plant had a better expression profile than the 2-year-old plant [1,26]. Consistent with these findings, our PCA also revealed differences in the metabolome and transcriptome profiles of 2-year and 3-year-old plants.

### 4.1. Metabolomic Analysis

Amino acids and derivatives constituted the major class of metabolites, with compounds in this class playing essential roles in antioxidant activities [27,28]. Further, the top up-regulated and down-regulated compounds were related to class nucleotide and derivatives, phenolic compounds, and lipids majorly, and these were found enriched in 3-year-old plants. These compounds have important implications in therapeutics. It has been demonstrated that the up-regulated metabolite methoprene acid ((2E, 4E)-11-methoxy-3,7,11-trimethyldodeca-2,4-dienoic acid) stimulates reporter genes for vertebrate RXR stimulation [29]. It has also been shown to have inhibitory effects on retinoid-regulated pathways [30]. The 9,10-Dihydroxy-12,13-epoxyoctadecanoic acid is another significant metabolite that has anti-inflammatory qualities and stimulates the synthesis of insulin and the absorption of glucose [31,32]. Ellagic acid (EA) is a naturally occurring secondary metabolite of bioactive polyphenolic compounds found in a wide variety of plant taxa. Ellagic acid (EA) has demonstrated anti-carcinogenic properties as well as actions that hinder the production of biofilms [33,34,35]. Pentacyclic triterpenoids such as ursolic acid and betulinic acid are both used in the treatment of non-communicable disorders [36]. The 2-Phenylethanol (2-PE) is a fragrant alcohol that has a scent similar to roses, and it is primarily produced via chemical synthesis and is extensively used in the food, cosmetic, and fragrance industry [37]. Arachidonic acid (AA) is an essential fatty acid. In both healthy and pathological circumstances, phospholipids in cell membranes produce AA, which is then processed by cyclooxygenase, cytochrome P450 enzymes, and lipid oxygenase pathways to control intricate cardiovascular function [38]. Moreover, a previous study reported that 5-Hydroxy 6,8,11,14-eicosatetraenoic acid is an omega-6 fatty acid that is structurally similar to arachidic acid and potentially involved in manufacturing vigorous lipid mediators that promote inflammation and could be crucial in inflammatory diseases like asthma [39]. Another metabolite, 8-Hydroxy-2-deoxyguanosine, was detected that serves as a potent biological biomarker for oxidative stress and carcinogenesis [40]. Previous research studies on mice indicated hydroxy linoleic acid as a bioactive lipid that exhibited anti-inflammatory attributes [41]. Phenylacetic acid amide is another metabolite that was previously involved in neuroprotective activities under focal brain ischemia conditions when experimented on male Wistar rats [42]. 4-O-glucosyl-sinapate is a phenolic acid possessing antioxidant activities in Proso millet [43].

The KEGG pathway analysis identified differentially enriched metabolites under different classes, such as lipids, phenolic acids, nucleotides, and their derivatives. Results revealed four metabolites from the lipids category were comparatively more highly enriched in PN3 than in PN2. The role of lipids, particularly hydroxyoctadecadionic acid (a derivative of linoleic acid), in the regulation of metabolic pathways linked to cancer and atherogenesis [44]. Moreover, 9- and 13-hydroxyoctadecadionic acid is potentially involved in essential processes of cell mitogenesis and apoptosis, which control programmed cell death and are essential for maintaining homeostasis in healthy cells, a mechanism that is often disrupted in cancer cells [45]. Chlorogenic acid is one of the most abundant polyphenolic compounds that has been detected in a variety of crop plants and was found to be highly enriched in the PN3 group compared to PN2. Previously, this compound has been suggested to have neuroprotective effects and has been highlighted in various studies to improve brain health and cardiovascular diseases [46,47]. Similarly, guanine and cytidine were also found to be highly enriched in PN3 compared to PN2. Previous studies reported the role of guanine-based purines in health-promoting activities as they are important in the central nervous system (CNS) as neuromodulators [48]. Moreover, cytidine derivatives were also found to have several medicinal applications, including antimicrobial and anticancer properties [49]. The differential metabolites and pathways identified in the present study have also been reported previously [50]. In addition, studies have suggested that serum homocysteine levels are associated with the metabolism of arachidonic acid and linoleic acid, which might play a role in homocysteine-induced vascular disease [51]. Similarly, nucleotide metabolism has been reported to be directly involved in regulating gene expression of purine and pyrimidine synthesis and has emerged as a significant pathway in immune cells and tumor cells, thus targeting nucleotide metabolism as a promising approach in immunotherapy [50]. The up-regulation of phytohormone signal transduction observed in the present study is an important finding as this pathway is crucial for plant defense response and plays a substantial role in growth, development, and environmental responses. It also stimulates cellular activity to reduce heavy metal stress and promote plant growth [52]. The differential enrichment of metabolites, particularly lipids and phenolic compounds, underscores age-related variations in metabolic pathways.

### 4.2. Transcriptome Analysis and Expression Pathways

A total of 3141 significantly differentially expressed genes identified in the present study were categorized mainly into cellular components, biological processes, and molecular functions, which is consistent with a previous study on *Radix gentianae*, a medicinal crop [53]. Further, the gene ontology enrichment analysis showed highly enriched pathways. For example, Heme proteins (typically responsible for binding heme cofactors) are involved in a multitude of biological processes such as electron transfer, oxygen transport, metal ion storage, chemical catalysis, gene expression, and cellular signaling [54]. Understanding the residues involved in heme binding sites can aid in deciphering the mechanism underlying heme-protein interactions and improving our comprehension of the biological roles of heme proteins. The expression level of antiapoptotic factors has recently been linked to a dire prognostic in acute myeloid leukemia (AML) and is extensively implicated in chemoresistance. So, altering apoptosis-related proteins has become a viable therapeutic approach [55].

The differentially expressed genes and metabolites identified in the present study were concurrently mapped to the KEGG pathway to better comprehend the interplay between metabolites and genes. Secondary metabolites (SM) are compounds that play a role in an organism’s interaction with its surroundings but are not essential to the life of the cell. These compounds frequently play a role in plants’ defense against biotic and abiotic stressors [56]. Secondary metabolites come from several families of metabolites and can be strongly induced in response to various stressors. In the previous study, the molecular evidence supporting the harvesting of *P. notoginseng* roots in the third year of growth was provided by the KEGG pathway analysis, which showed that the roots of the three-year-old plant had less activity in primary metabolism, cell growth, and differentiation. However, secondary metabolisms were more active [57]. The current study also revealed higher expression of plant secondary metabolites and up-regulation of genes in these pathways in 3-year-old *P. notoginseng*. The production of mugineinc acid via the up-regulation of two genes, *MAT* and *DMAS*, has been previously linked with Fe deficiency response [58,59]. Pentagalloylglucose is related to ellagitannin metabolism [60]. Pentagalloylglucose, a hydrolyzable tannin found naturally in many traditional medicinal plants, is an active molecule with a variety of biological actions [61,62]. The biosynthesis of pentagalloylglucose was up-regulated by two genes, shikimate dehydrogenase (*SDH*) and gallate 1-beta-glucosyltransferase genes in the shikimate pathway as described previously [63]. Coumarins are non-cyanogenic defense compounds that are stored as β-D-glucosides, which are hydrolyzed by specific β-glucosidases [64]. The coumarins exhibit many biological functions, such as anti-inflammatory, antioxidant, anticancer, antiviral, anticoagulant, and more. They thus have considerable promise as pharmaceuticals [65].

All aspects of plant growth, development, and immunity depend on phytohormones; nevertheless, the control of these processes is primarily determined by the interactions between phytohormones, which vary dynamically during these processes. The specificity of the interaction, such as the plant/pathogen species, the kind of tissue/organ, the degree of the stress, and the response timescale studied, determines how much phytohormones contribute to abiotic and biotic environmental challenges [66]. The current study displayed down-regulation of the *AUX1* gene while up-regulation of *SAUR* in the auxin signaling pathway. Auxin, a plant hormone, controls several aspects of plant growth and development. Auxin is predominantly produced in the developing leaf primordia and the apex of the shoot. It is then transferred to target tissues, like the roots, and its transport is mediated by carriers such as the *AUX1* gene family (major auxin influx carriers) [67]. In a previous study, the role of *AUX1* auxin influx carriers in regulating Arabidopsis development was examined, indicating their involvement in various biological processes, including seed germination, root, shoot, flower, and embryogenesis development [68]. The *SAUR* (small auxin-up RNA) genes constitute the largest family of early auxin-responsive genes and have been investigated in many plant species, including *Arabidopsis thaliana*, *Zea mays*, and *Oryza sativa*. Up until now, it has been demonstrated that several *SAUR* genes are involved in various aspects of plant development, growth, and stress response [69]. Two genes, *CRE1* and *AHP*, were down-regulated in the cytokine pathway; however, the down-regulation of *CRE1* in a previous study suggested that it was a contributing factor in decreasing cell division and root initiation [70].

The cytokinin *CRE1* pathway affects root development and tolerance to biotic and abiotic environmental challenges, and it is also necessary for symbiotic nodule organogenesis, according to a previous study on *Medicago truncatula* [71], while AHP genes regulate vegetative growth and other developmental activities in plants [72]. Another gene, *GID1*, was up-regulated in the gibberellin signaling pathway, and *PP2C* was up-regulated in the abscisic acid signaling pathway. Gibberellin (GA) is involved in the control of biotic and abiotic stress responses in plants, as well as their growth and development. Studies reported that the N-terminal extension of *GID1* undergoes a conformational change upon binding of bioactive GA to the GA-INSENSITIVE DWARF1 (*GID1*) receptor. This conformational change facilitates the interaction of the complex GA-GID1 with DELLAs and is involved in regulating plant growth [73]. The *PP2C* genes negatively regulate ABA signaling and play an important role in growth, development, and stress signal transduction in plants [74]. Moreover, four genes, including *ETR*, *CTR1*, *EBF1/2*, and *ERF1/2*, were up-regulated in the ethylene signaling pathway, and *SIMKK* was down-regulated, and these results validated the previous research on tea plants [75]. Ethylene (ETH) is actively involved in fruit ripening, plant growth, senescence induction, seed germination, and the development of resistance in plant-pathogen interactions [76]. In the brassinosteroid signaling pathway, three genes, including *BZR1/2*, *TCH4*, and *CYCD3*, were down-regulated. Studies have suggested that *BZR1/2* could bind to particular regions on downstream target gene promoters, thereby enhancing disease resistance by activating the up-regulated expression of *TCH4* and *CYCD3* genes. The *TCH4* gene is essential for biological functions in plants, including disease resistance, cell elongation, and stress adaptation [77]. Further studies have revealed that the *CYCD3* gene, which is involved in the BRs cell signaling pathway, is linked to cell division [78]. Further, *JAR1* was down-regulated in the jasmonic acid signaling pathway, and *NPR1* and *PR-1* were down-regulated in salicylic acid signaling pathways. Moreover, an essential function of the nonexpressor of pathogenesis-related 1 (*NPR1*) is to facilitate signaling pathways associated with plant immunity and resistance to abiotic stress. More specifically, it has been determined that the *NPR1* protein plays a critical role in the regulation of broad-spectrum, long-lasting systemic acquired resistance (SAR), which enhances resistance to pathogens such as bacteria, viruses, and fungi. Numerous pathogenesis-related (PR) genes are triggered during SAR, both systemically in distant plant tissues and locally at the infection site [79]. These findings collectively indicate that in the *P. notoginseng* plant, different genes were up-regulated with age, involving different biological pathways, including the metabolism of lipids (linoleic and arachidonic acids metabolism), nucleotides, hormone signaling, plant growth, pathogen, and stress resistance. The metabolites produced as a result of these metabolic pathways resulted in a better phytochemical profile of roots, leading to promising therapeutic potential as the chlorogenic acid and ginsenoside contents in mature roots were quite higher compared to younger ones, as reported previously [14].

## 5. Conclusions

This study concluded that roots of 3-year-old *P. notoginseng* had better metabolome and transcriptome profiles than 2-year-old plants, which is mainly attributed to the up-regulation of important genes and metabolic pathways. The 3-year-old roots clearly showed up-regulation of pathways related to nutrient metabolism (mainly lipids and nucleotide metabolism), production of secondary metabolites, and signal transduction. These findings concluded that with advancing age, various biological processes are up-regulated to meet requirements of plant growth and protection against stress and diseases via accelerating nutrient metabolism and accumulation of secondary metabolites. These findings provide deeper insights into the plant’s dynamic biochemical and molecular changes during its growth that could have implications for its therapeutic uses owing to enriched metabolite contents.

## Data Availability

Data are contained within the article.

## Supplementary Materials

The following supporting information can be downloaded at: https://www.mdpi.com/article/10.3390/plants13111441/s1, Figure S1: Correlation of metabolome profiles of PN2 vs. PN3 replicates; Figure S2: Cluster analysis of metabolome of PN2 vs. PN3; Figure S3: The boxplot of the FPKM distribution of each sample; Figure S4: Correlation of transcriptome profiles of PN2 vs. PN3 replicates; Table S1: The sequencing statistics for 6 RNA libraries of *P. notoginseng*.
