# Supplementary material for: Comparative Metabolome and Transcriptome Analyses Reveal Differential Enrichment of Metabolites with Age in Panax notoginseng Roots
Source: Plants (Basel). 2024 May 23;13(11):1441. doi: 10.3390/plants13111441 (PMC11175106; doi:10.3390/plants13111441)
Supplement: Supplementary file 1 [file plants-13-01441-s001.zip › plants-2939124-supplementary.pdf]

Supplementary file

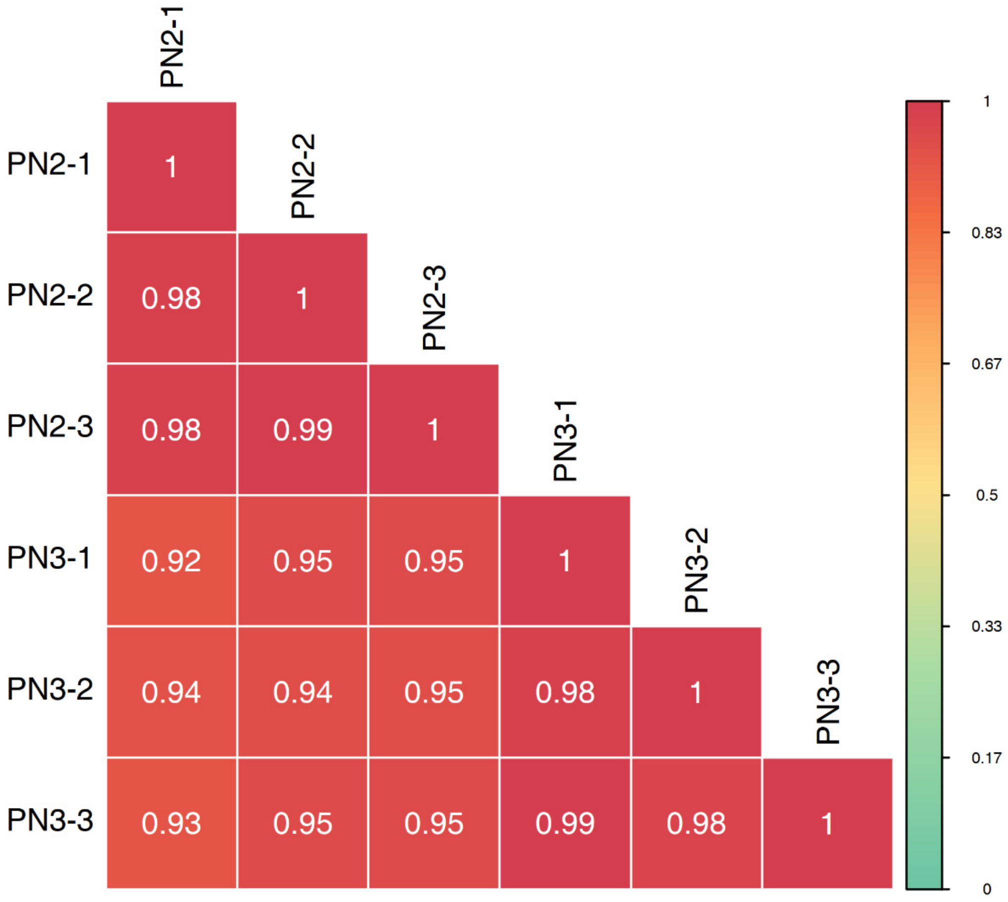

Figure S1. Correlation of metabolome profiles of PN2 vs PN3 replicates.

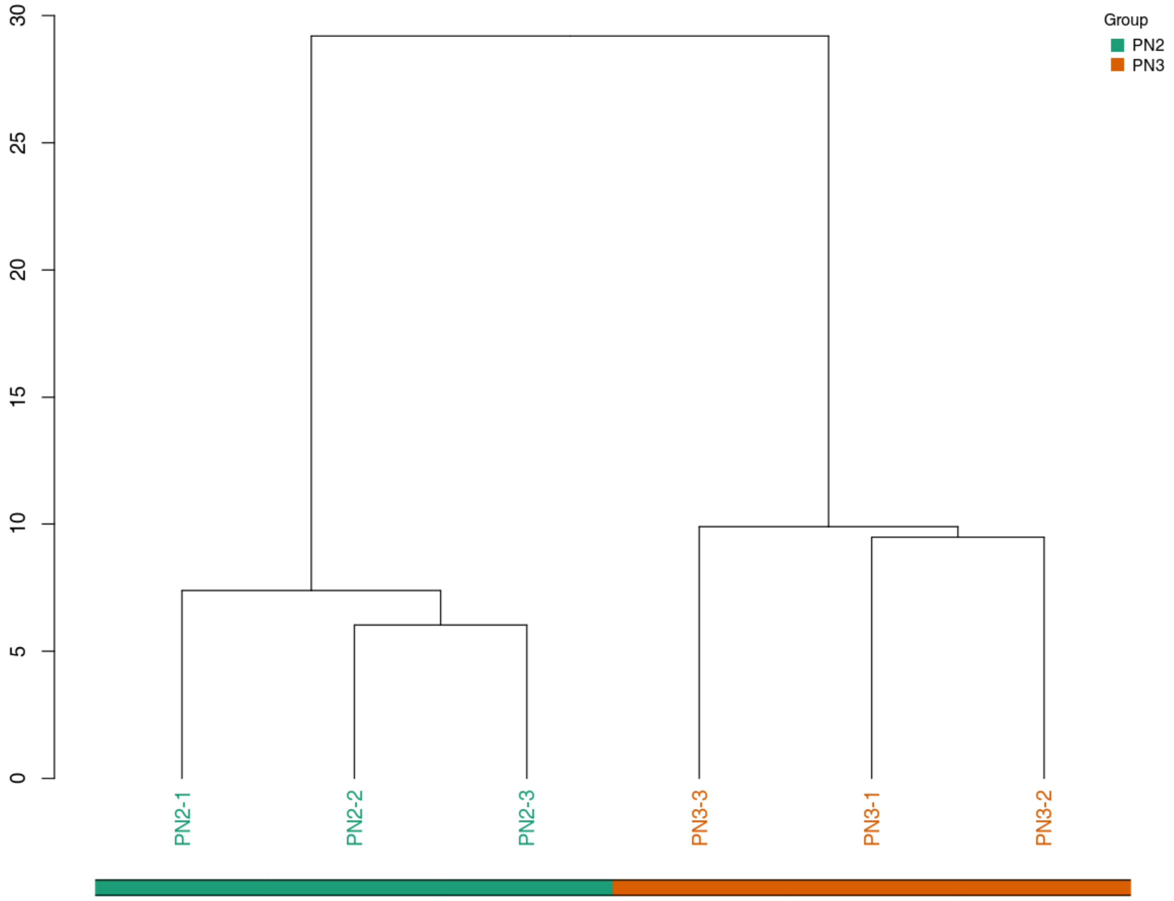

Figure S2. Cluster analysis of metabolome of PN2 vs PN3.

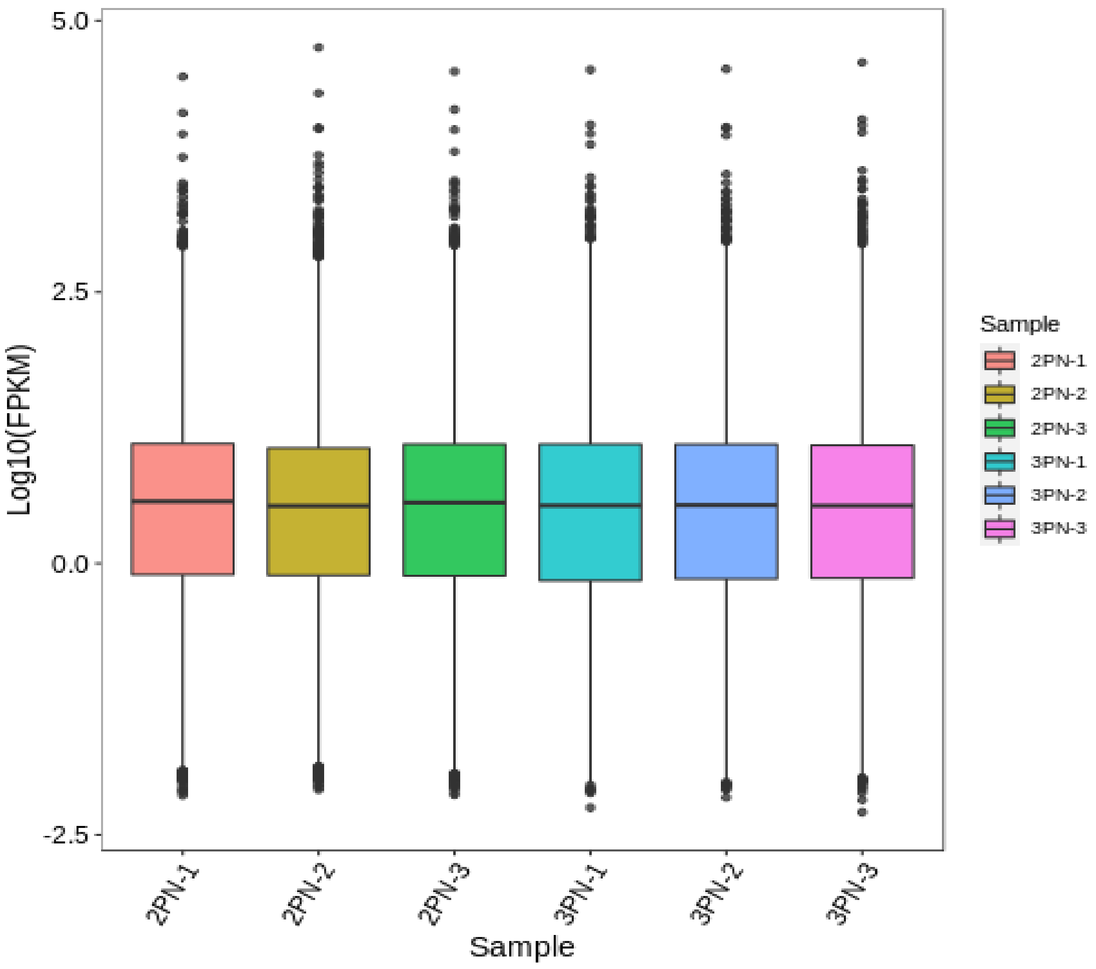

Figure S3. The boxplot of the FPKM distribution of each sample.

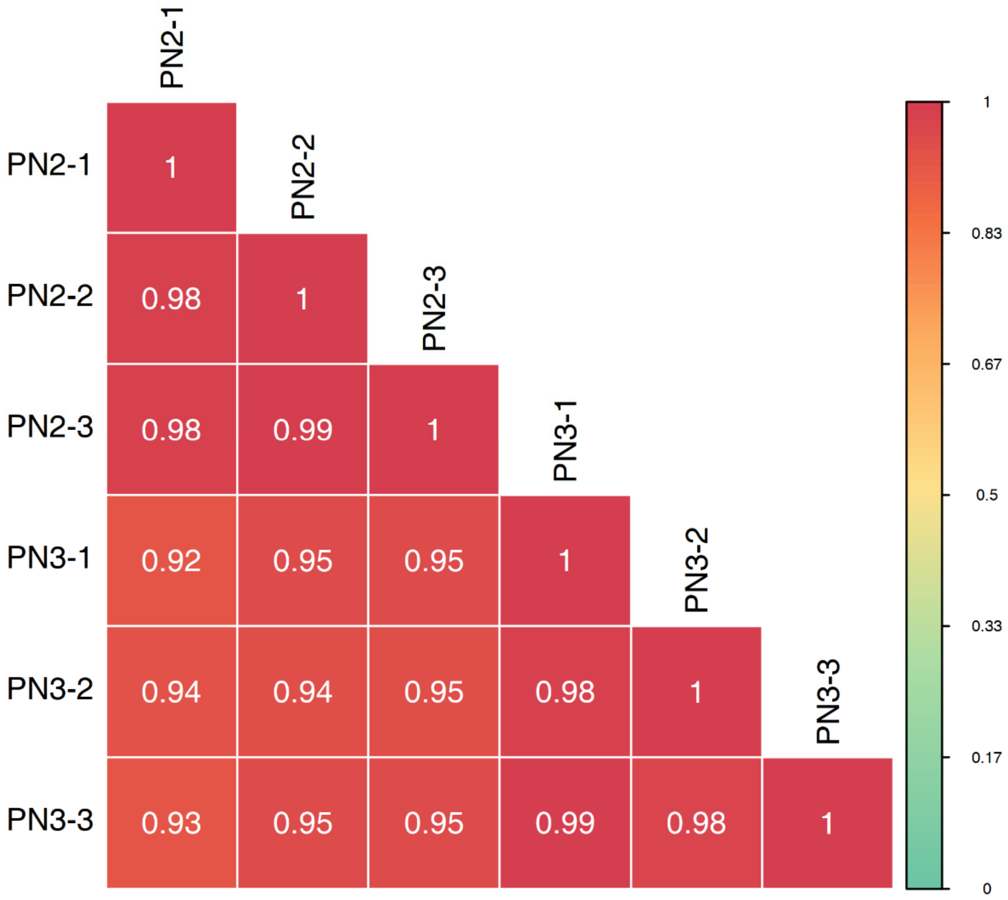

Figure S4. Correlation of transcriptome profiles of PN2 vs PN3 replicates.

Table S1. The sequencing statistics for 6 RNA libraries of *P. notogensing*.

Sample	Raw Reads	Clean Reads	Clean Base(G)	Error Rate(%)	Q20(%)	Q30(%)	GC Content(%)
2PN-1	42913520	41513802	6.23	0.03	97.93	93.72	43.28
2PN-2	45351936	42456072	6.37	0.03	98.07	94.09	43.32
2PN-3	43956592	42503406	6.38	0.03	98.09	94.26	43.52
3PN-1	47002482	45574112	6.84	0.03	97.93	93.93	43.69
3PN-2	42271528	40500642	6.08	0.03	98.04	94.21	43.66
3PN-3	43597894	41523342	6.23	0.03	98.06	94.29	43.43
Average	44182325	42345229.33	6.355	0.03	98.02	94.08333	43.48333
